# Intelligent Spacecraft Visual GNC Architecture With the State-Of-the-Art AI Components for On-Orbit Manipulation

**DOI:** 10.3389/frobt.2021.639327

**Published:** 2021-06-01

**Authors:** Zhou Hao, R. B. Ashith Shyam, Arunkumar Rathinam, Yang Gao

**Affiliations:** Surrey Space Center, University of Surrey, Guildford, United Kingdom

**Keywords:** space robotics, artificial intelligent, Guidance Navigation and Control, on-orbit service, space manipulator, pose estimation

## Abstract

Conventional spacecraft Guidance, Navigation, and Control (GNC) architectures have been designed to receive and execute commands from ground control with minimal automation and autonomy onboard spacecraft. In contrast, Artificial Intelligence (AI)-based systems can allow real-time decision-making by considering system information that is difficult to model and incorporate in the conventional decision-making process involving ground control or human operators. With growing interests in on-orbit services with manipulation, the conventional GNC faces numerous challenges in adapting to a wide range of possible scenarios, such as removing unknown debris, potentially addressed using emerging AI-enabled robotic technologies. However, a complete paradigm shift may need years' efforts. As an intermediate solution, we introduce a novel visual GNC system with two state-of-the-art AI modules to replace the corresponding functions in the conventional GNC system for on-orbit manipulation. The AI components are as follows: (i) A Deep Learning (DL)-based pose estimation algorithm that can estimate a target's pose from two-dimensional images using a pre-trained neural network without requiring any prior information on the dynamics or state of the target. (ii) A technique for modeling and controlling space robot manipulator trajectories using probabilistic modeling and reproduction to previously unseen situations to avoid complex trajectory optimizations on board. This also minimizes the attitude disturbances of spacecraft induced on it due to the motion of the robot arm. This architecture uses a centralized camera network as the main sensor, and the trajectory learning module of the 7 degrees of freedom robotic arm is integrated into the GNC system. The intelligent visual GNC system is demonstrated by simulation of a conceptual mission—AISAT. The mission is a micro-satellite to carry out on-orbit manipulation around a non-cooperative CubeSat. The simulation shows how the GNC system works in discrete-time simulation with the control and trajectory planning are generated in Matlab/Simulink. The physics rendering engine, Eevee, renders the whole simulation to provide a graphic realism for the DL pose estimation. In the end, the testbeds developed to evaluate and demonstrate the GNC system are also introduced. The novel intelligent GNC system can be a stepping stone toward future fully autonomous orbital robot systems.

## Introduction

With the increasing interest in orbital robotic manipulation for on-orbit servicing missions, the future Guidance, Navigation, and Control (GNC) systems are inclined to implement intelligent on-board systems with greater autonomy to address the challenges associated with the time-critical and highly unpredictable scenarios. To clarify the definition of GNC for future space robotic systems, we have included the manipulation of the robotic arms together with the conventional spacecraft GNC system, such as the Attitude and Orbit Control System (AOCS). Both these systems should be seamlessly integrated in order to fully utilize all available “sensing” information, which is essential to deliver intelligent solution to the mission operations.

Conventional optimization and control methods have evidenced great results for on-orbit manipulation with rendezvous and docking in renowned mission scenarios (Virgili-Llop and Romano, 2019). These achievements are based on the accumulated work to study the service spacecraft's dynamics and kinematics with robotic arms in the free-floating condition (Umetani and Yoshida, 1989; Dubowsky and Papadopoulos, 1993; Wilde et al., 2018). The coupled dynamics of the spacecraft and manipulator system make the planning and control problem extremely difficult and computationally expensive. This makes the free-floating mode less useful and risky for real space missions. To date, all of the recent research on planning and control of free-floating spacecraft are limited to simulation and laboratory environments.

Using Artificial Intelligence (AI) technology in the GNC systems provides the opportunity to carry out real-time on-board decision-making in an unforeseen and a time-critical situation with the autonomous optimization of the on-orbit manipulation for the both co-operative and non-cooperative targets. More accurately, in this research, AI refers to machine learning. Machine learning, especially Deep Learning (DL), has depicted tremendous progress and achievement to resolve complicated optimization problems in robotics (Goodfellow et al., 2016). Two specific fields are useful for spacecraft GNC systems: (1) Learning-based Computer Vision (CV) algorithms that determine an object's 6 degrees of freedom (DOF) information (ESA, 2019b). (2) Learning-based methods that solve non-linear multi-body planning and control problems (Pierson and Gashler, 2017). Both fields are active, and some of the recent research use simulation and on-ground experiments to demonstrate the feasibility of using the relevant space applications.

Despite the huge potential the emerging AI technique shows in improving the autonomy and efficiency of the GNC system, to the best of knowledge of the authors, there are no missions that use AI for GNC-related tasks. The space industry already realized these benefits and aimed to introduce AI to space, especially for the GNC systems. Many foresee the future with fully intelligent GNC spacecrafts, which organically carry out objectives without human intervention that has been widely discussed (Truszkowski et al., 2009; Starek et al., 2016; Nanjangud et al., 2018). However, developing and using such a fully intelligent system for space is still considered far-fetched because of the reality of the low Technology Readiness Levels (TRLs) for both hardware and software and the unsettled questions in the associate laws and regulations in AI.

Here, we show an intermediate and novel solution as a conceptual architecture to replace some of the conventional functional blocks with state-of-the-art AI algorithms for CV and robotic arm trajectory planning. The authors believe the major contribution of this research is the introduction of a novel intermediate intelligent visual GNC system with state-of-the-art AI components for spacecraft with robotic arms. The AI components are as follows:

- A DL-based method to exploit two-dimensional (2D) images for target identification and close-proximity range relative guidance and navigation.
- A computationally inexpensive learning-based motion planning algorithm for free-floating spacecraft with robotic arms.

This paper is organized as follows: section Introduction gives the introduction regarding the background information about the research. In section The Intelligent GNC System Design, we provide the overview of the proposed visual GNC system architecture and hardware design. Sections Deep-Learning Method For Target Pose Estimation, and section Learning-Based Method For Robotic ARM Manipulation and Control convey the detailed design of the two AI components, respectively: a DL-based target identification and a pose estimation method based on 2D images and a learning-based motion planner for free-floating space manipulation with the optimization about spacecraft's attitude. These two state-of-the-art AI packages can potentially increase the autonomy of on-orbit service missions that comprises manipulation. In section Testbed Setups and The Demonstration Missiion, a demonstration mission to showcase the proposed AI GNC system is presented. The mission is to capture a 16U malfunctioning CubeSat by using a service spacecraft with a 7-DOF robotic arm. The testbeds and a detailed simulation are exemplified and explained. The benefits and limitations of the AI GNC system are also discussed in the end of this section. Finally, in section Conclusion and Future Works, we conclude the study discussing various ongoing developments and direction for future research.

## The Intelligent GNC System Design

### Overview of the Intelligent Service Spacecraft

The main spacecraft platform used for this concept development and simulation is similar to the Surrey Space Center's RemoveDebris microsatellite (Forshaw et al., 2016). Besides, it has a 7-DOF articulated robotic arm attached for custom manipulation tasks. The spacecraft's primary task is to detect, approach, and service orbiting targets. The technology and hardware are all considered as scalable. The proposed AI GNC system is based on this hardware design with the essential subsystems that include:

- A main monocular camera for navigation and 360° sub-cameras for no dead-zone awareness,
- A 7-DOF redundant robotic arm,
- Onboard computational hardware suitable for CV.

**Figure 1:**
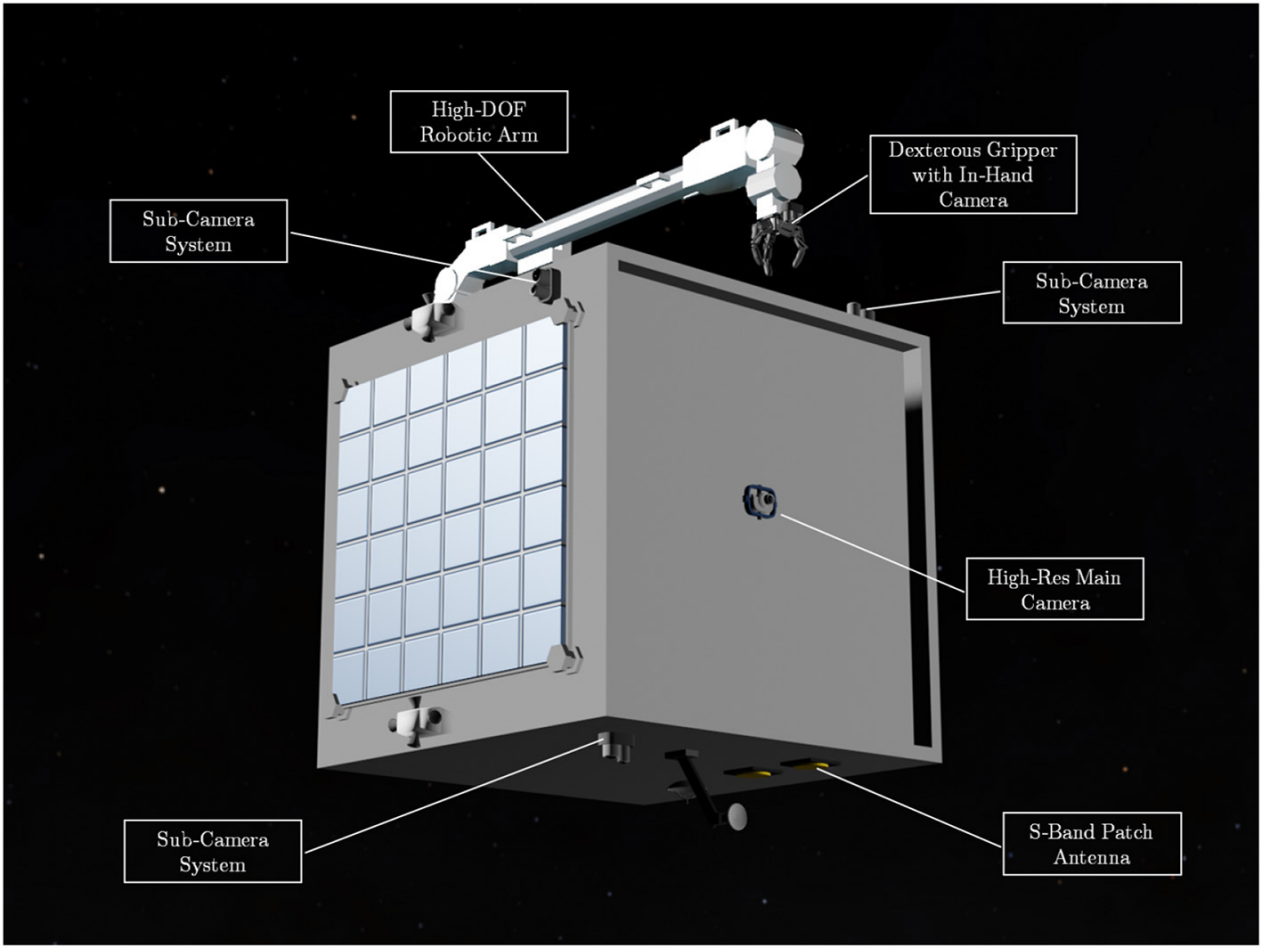
The design of the intelligent orbital service spacecraft—AISAT: it comprises a monocular camera network and a 7-DOF (degrees of freedom) robotic arm as key components for the intelligent visual Guidance, Navigation, and Control (GNC) subsystem. The spacecraft also has a dexterous gripper for target capture and S-band patch antenna for basic house-keeping and the intelligent system updating.

**Figure 2:**
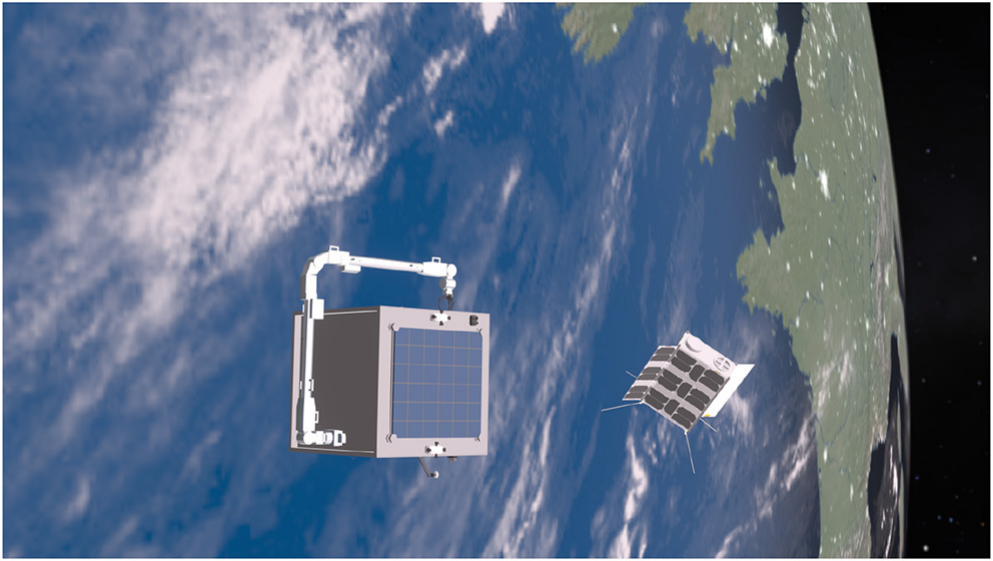
The conceptual mission for capturing and servicing a non-cooperative but known 16U CubeSat.

The system design of the spacecraft is illustrated in Figure 1. The core concept is that the proposed intelligent AI-based GNC subsystem uses the information provided by visual sensors to carry out pose estimation and motion planning of robot arm. Visual sensors including monocular cameras are no strangers to space missions as they have been widely used as payloads and sensors for Attitude Determination and Control System (ADCS), such as star tracker and Naidr sensor. The proposed AI GNC system uses monocular cameras for the visual feedback including target identification and pose estimation. Monocular camera approaches are relatively new and the cost-effectiveness makes it more attractive for space missions than stereo cameras and LiDARs, which have been demonstrated for the relative navigation for space berthing and docking (Opromolla et al., 2017). The monocular camera network proposed here consists of five sub-camera systems located on each face of the microsatellite bus. Each sub-camera system has a 50 mm (telescope) and an 18 mm (wide) lens with 120° Field of View (FOV) to cover both mid- to long-range and close-proximate range targets detection and pose estimation. These monocular cameras could also be used as sun sensors and star tracker. The main camera locating at the front-facing to the nominal orbiting direction of the spacecraft is a single Hi-Res wide lens camera to maximize the image quality in pixel clarity for long-range orbiting target identification and pose estimation. The complete camera network provides a 360° coverage for no dead-zone awareness. As for the relative navigation algorithms, DL-based methods are the new trend to replace marker-based methods, which usually suffer from reduced robustness in harsh lighting conditions (Pasqualetto Cassinis et al., 2019). The 7-DOF articulated robot arm in the combined AI GNC system is inspired by the European Robotic Arm (ERA) (Boumans and Heemskerk, 1998). High-DOF robotic arms are more challenging to operate but offer redundancy to generate wider trajectory options for machine learning. Overall, Figure 2 illustrates the proposed mission at its close-proximity operation range after the orbit rendezvous finished. The mission aims to demonstrate the AI components and show the feasibility of the intelligent GNC system. For safety concerns, the mission design still consider ground-based interventions involved. Therefore, the ground communication should remain stable during the manipulation.

### The Intelligent GNC System Architecture

**Figure 3:**
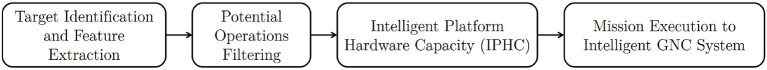
The decision-making flow chart for the intelligent Guidance, Navigation, and Control (GNC) system. The achievable tasks are based on the hardware constraints and the task is selected based on the target's identify and features.

The intelligent space missions envision a top-level and real-time decision-making process for unforeseen events from pre-trained knowledge. The identification of the target is the minimum information that is required for the intelligent GNC system to plan and execute the corresponding actions. The AI GNC system introduced in this article uses the identification of the target for defining the mission and relative orbit navigation. Nevertheless, the proposed intelligent GNC system differs from the existing concept of spacecraft autonomy that focuses on orbital determination and control, such as task planning and re-planning for Earth observation, communication relays, and formation flying. Their primary objectives are predefined, and the performance is optimized and merited with existing models to be executed automatically in space (Tipaldi and Glielmo, 2018). The conventional spacecraft mission analysis is usually carried out on the ground or in-space if astronauts are involved. In contrast, the intelligent system could make decisions onboard and the task the spacecraft can carry out constraint by the Intelligent Platform Hardware Capacity (IPHC) of the space platforms. Table 1 shows the IPHC of the essential subsystems for the achievable intelligent space missions. The automatic task planning and re-planning with multiple decision-making algorithms could be potentially used as the backbone of the intelligent decision making, for example, the methods discussed by Chien et al. (2005). Figure 3 shows the ideal on-board intelligent decision-making flow chart. However, high-level intelligent decision-making is still considered of high risk; thus, the stable ground communication that can monitor and intervene the mission is preferred as an intermediate solution.

**Table 1:** Intelligent Platform Hardware Capacity (IPHC) links the combination of robotic hardware with the potential type of missions.

	**Robot manipulator**	**Robotic sensor**	**Robot connector/Gripper**
In-space assembly	Essential	Essential	Essential
On-orbit servicing	Essential	Essential	Essential
Active Debris removal	Non-essential	Essential	Non-essential
Intelligent orbit planning and maneuver	N/A	Essential	N/A
Autonomous berthing and docking	Non-essential	Essential	Essential

**Figure 4:**
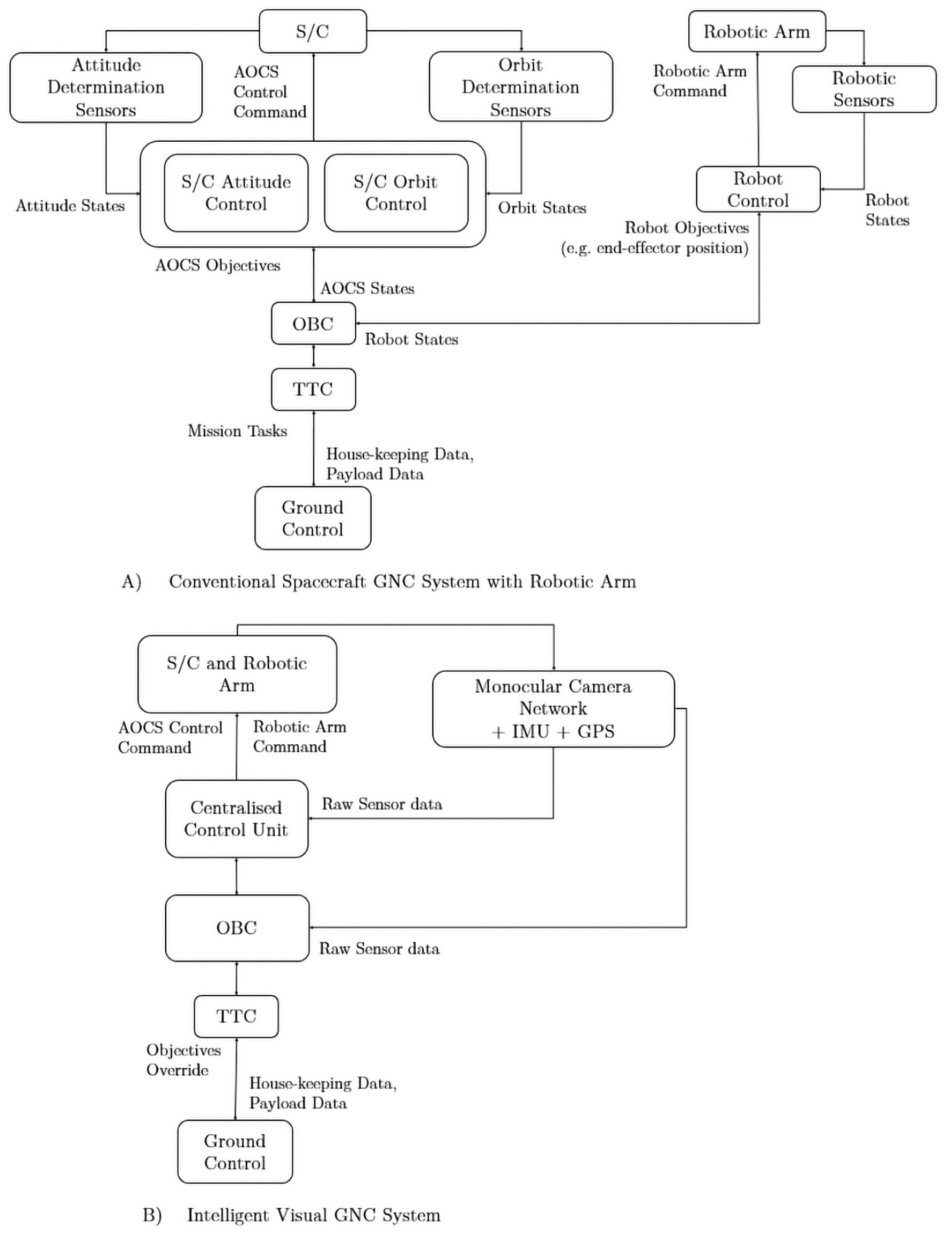
The transition from **(A)** conventional spacecraft Guidance, Navigation, and Control (GNC) subsystem with robotic arm to **(B)** the Artificial Intelligence (AI) GNC subsystem. The AI GNC system uses centralized sensor information with primarily 2D images as inputs to the AI computer with pre-trained neural networks to control the spacecraft and robotic arm at the same time.

To picture the differences, the system flowcharts in Figure 4 show the comparison of the conventional spacecraft GNC systems and the proposed intelligent GNC spacecraft. The intelligent GNC system can determine the target states and perform orbital maneuver, attitude control, and robotic arm control adaptively to the situation *in situ* without the needs of ground commands. The system can off-load the pressure for the on-board telecommunication system to conserve energy and reduce the cost associated with the ground operations, which are inevitable for the conventional GNC systems. Furthermore, in contrast to the conventional GNC systems where the control systems are usually managed by different subsystems with limited data exchange and decision-making, the intelligent GNC system can exploit all sensor information to use pre-trained neural networks to deliver the optimized control results. Such a system demands the standardized communication networks across all sensors, and a more powerful logic unit for handling raw sensor data and processing globally optimized control schemes to simultaneously manage orbital control, spacecraft attitude control, and the robotic arm control. From this comparison, the major technical challenges for the intelligent GNC architecture have been identified are as follows: (1) low computational power hardware in space radiation environment and limited energy supply; (2) robustness of the pre-trained neural networks for real space missions. The first challenge is less problematic for LEO missions as the commercial-off-the-shelf (COTS) components could be used with limited radiation coating and the performance of the radiation-resistant DSP/FPGA computers are also improving. The second challenge is more general for any AI-based system. The way to address this challenge is to provide more robust and divergence data for the network training and carrying out extensive testings before flight.

## Deep-Learning Method for Target Pose Estimation

This section discusses the relative pose estimation of the target spacecraft or object using a monocular camera and a keypoint-based DL framework. In orbital robotic missions involving autonomous close proximity operations, the primary information required is the knowledge on the relative state of the chaser spacecraft with respect to the target object/spacecraft or vice versa. To perform relative navigation, a multitude of sensor options available to gather necessary input information. However, vision-based navigation using monocular cameras is more preferred than other approaches because of its simplicity, low power consumption, and availability of space hardware at a comparatively lower price. Monocular cameras are traditionally used to perform autonomous spacecraft guidance and navigation in the far-range, while in the mid- and close-range rendezvous operations the ground control is usually kept in the loop to perform any major operations. The space imagery is characterized by high-contrast images with sharp changes in shadows, and low signal-to-noise ratio makes vision-based navigation in space extremely difficult, thus making the traditional feature-based or model-based approaches vulnerable to false state estimates or high computational requirements. The rise of DL has pushed the boundaries of image classification and image segmentation tasks in the field of CV to a near human-level accuracy. The growth of hardware and the availability made the DL tools readily available for research activities that pushed the algorithms to more robust and efficient.

The use of DL for pose-estimation has been around for some years in robotics, whereas in space, it is relatively new and evolving. In recent years, there is a growing interest in achieving autonomous visual navigation in orbit in this process many research works evolved in the field of DL-based pose estimation for spacecraft relative navigation. Some of the important research works are summarized below and this includes methods that offer state-of-the-art performance in benchmark datasets. Sharma and D'Amico (2019) proposed Spacecraft Pose Network (SPN) using a combination of classification and regression approaches to computing the relative pose. Chen et al. (2019) proposed estimation of the relative pose with deep landmark regression, where it involves both object detection and 2D landmark regression. Proenca and Gao (2020) proposed a hybrid approach for pose estimation and the network architecture has two branches with one dedicated for location and the other for orientation estimation. A simple regression was used for location estimation, and the probabilistic soft classification was used for orientation estimation. Gerard (2019) proposed a segmentation-driven approach to computing the pose of the target object.

**Figure 5:**
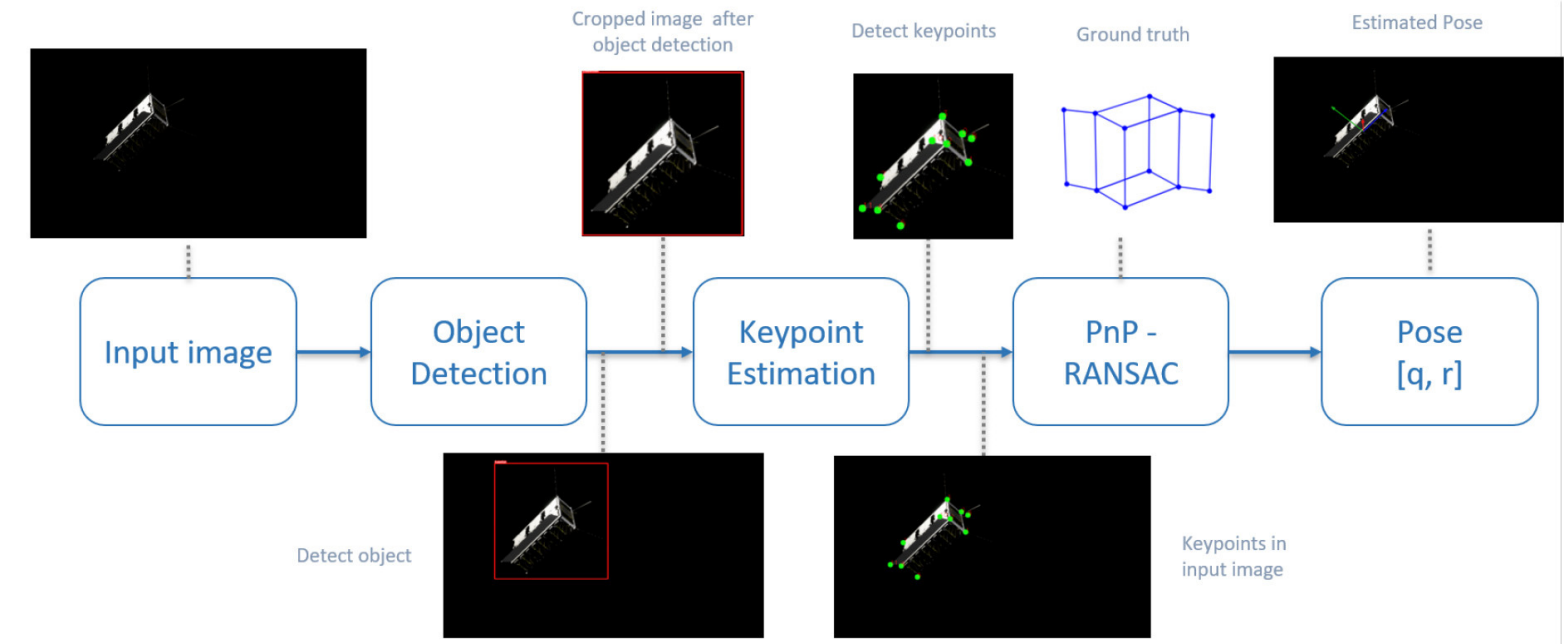
Sample photo-realistic visuals of CubeSat model in orbit generated using the OrViS simulator.

To train a DL model, it is essential to have a good quality dataset. Acquiring the space imagery of the target is either expensive or in most cases datasets with labeled ground truth is not readily available, and the viable alternative is to generate synthetic datasets using the photo-realistic simulators. We developed a visual simulator based on the state-of-the-art game engine Unreal Engine 4 (UE4), named OrViS (Orbital Visual Simulator), and it allows obtaining photo-realistic images and depth masks of the target in orbit. The details of the simulator were presented in Proenca and Gao (2020) and the sample images of the target CubeSat in orbit generated from the OrViS simulator are shown in Figure 5.

**Figure 6:**
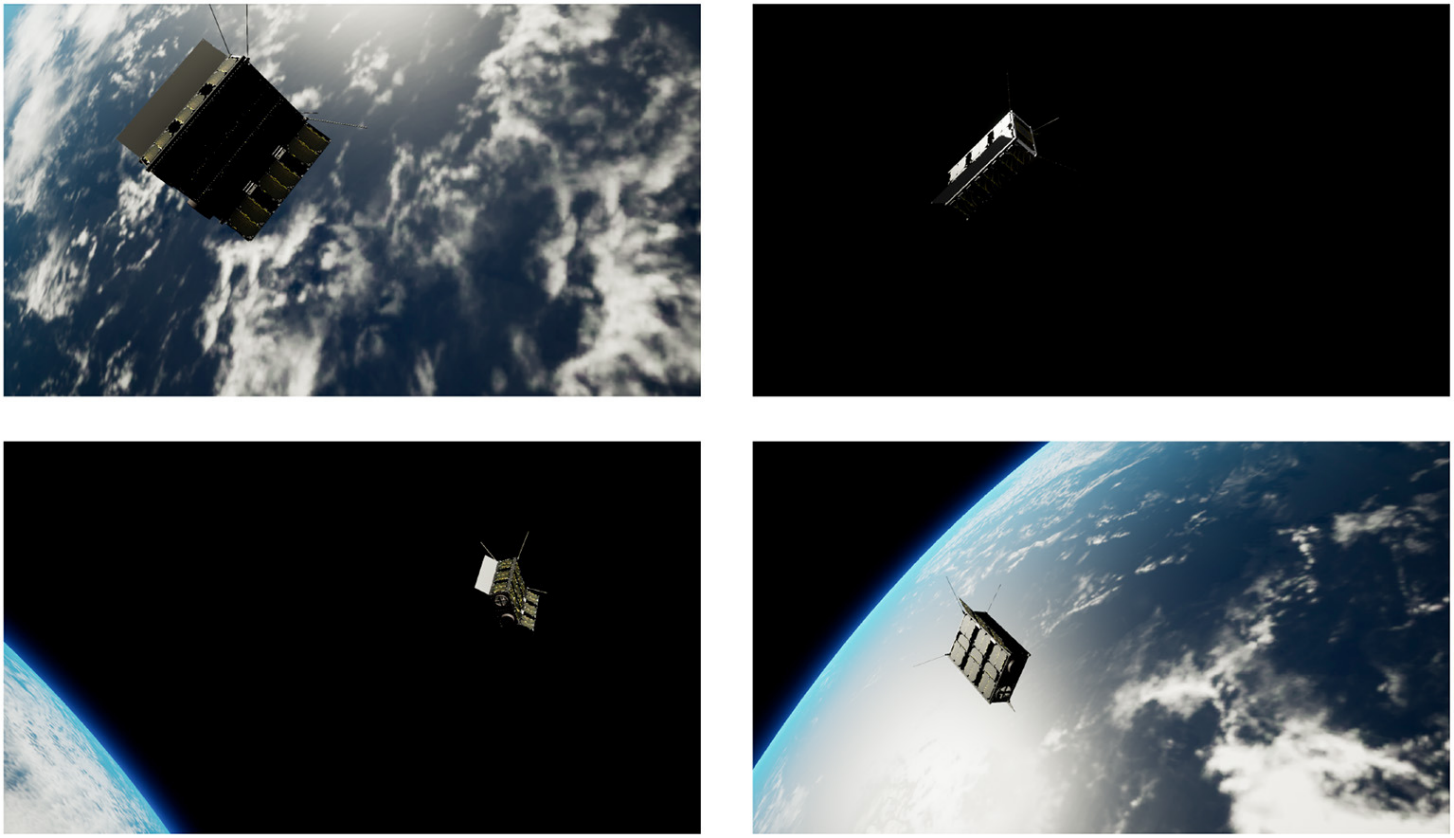
Framework for spacecraft pose estimation using keypoint-based deep learning approach.

To perform relative pose estimation for rendezvous with the non-cooperative spacecraft, we developed two approaches, non-keypoint-based approach presented in Proenca and Gao (2020) and keypoint-based approach presented in Rathinam and Gao (2020). A keypoint-based framework is discussed in this work because it offers better performance and accuracy. The keypoint-based pose estimation framework is shown in Figure 6 and it involves three major steps: they are object detection, keypoint-estimation, followed by perspective-n-point (PnP) projection for the pose computation. To perform object detection, a faster R-CNN using ResNet-50 architecture as a backbone was trained using the transfer learning approach. During the training process, the models were loaded with the pre-trained models from COCO dataset and the last few layers of the network were fine-tuned to identify the bounding box locations of the target CubeSat. The networks were trained on one NVIDIA Quadro P4000 8GB GPU. The training parameters include the input image size of 320 with a batch size of 8, a learning rate of 5*e*^−3^, SGD with a momentum of 0.9, and a weight decay regularization of 5*e*^−4^. Input image augmentations, such as random rotation, random translation, coarse dropouts, Gaussian noise, random brightness, and contrast were randomly added to make the training model more robust. The training converges quickly around 30 epochs for both the models.

The second part of the framework is keypoint estimation, which involves 2D landmarks regression and it uses the HigherHRNet network from Cheng et al. (2020). The network was modified to train the 12 keypoints of interest that are located around the 6U CubeSat. During the training process, the model was trained using the cropped images from the ground truth bounding box locations (initially used for the object detection) and the input data includes the cropped image along with the keypoints locations and their corresponding point visibility. The network architecture we used has 32 channels in the highest resolution feature maps and provides an output at two different scales, 1/4 and 1/2. The input image size was set to 512, which results in the output heatmap sizes of 128 and 256, from which we can infer the keypoint locations. An Adam optimizer is used with a learning rate of 1*e*^−3^ and momentum of 0.9. Image augmentation performed on the input image includes random rotation ([−30°, 30°]), random translation up to ([−30%, 30%]), coarse dropouts, Gaussian noise, random brightness, and contrast. For keypoint regression, the training time is quite extensive and the network provides required performance after 300 epochs of training and the image augmentation plays a key role in identifying the right keypoints. With the known 2D and three-dimensional (3D) correspondences, PnP algorithm is used to compute the camera pose. To avoid the false correspondences among the derived keypoints, and to eliminate the outliers, a RANSAC-based outlier rejection is also used while estimating the camera pose.

A brief analysis of the algorithm's performance in comparison to the state-of-the-art DL-based spacecraft pose estimation methods using different datasets is presented in detail in Rathinam and Gao (2020). The algorithm will be extensively tested and validated on the re-configurable orbital robotics testbed at the University of Surrey using the real CubeSat model with the lighting conditions similar to that of space. The hardware testing process will include using commercially off-the-shelf sensor components and low-power computing board (such as Nvidia Jetson) similar to perform DL-based state estimation on-board the spacecraft.

## Learning-Based Method for Robotic Arm Manipulation and Control

We propose a robot arm having 7 DOF. The main reasons behind this proposal are as follows:

1. The presence of the redundant DOF ensures multiple trajectories from the same start to the same target pose and an optimality criteria (min power, min attitude disturbance, etc.) can be set to chose a trajectory.
2. Possible to carry out a variety of tasks just like a human arm.
3. Software control remains the same, whereas hardware (like dimensions of the links, motor max torque) can be different depending on the task (like assembly or debris removal).

It is known that computationally expensive control strategies are largely discouraged in space. Hence, a learning-based trajectory generation technique is detailed, which learns the trajectories offline by encoding trajectories as a Gaussian probabilistic distribution. The trajectories can be reproduced by sampling and conditioning the Gaussian. We use a method called Probabilistic Movement Primitives as described in the work of Shyam et al. (2019). The two main differences between Shyam et al. (2019) and the current work are as follows:

- A model predictive controller (MPC) is used to generate trajectories instead of human demonstrations,
- Incremental learning to improve accuracy.

Expert human demonstrations is capable of producing jerk-free trajectories, but in this work, MPC is preferred because micro-gravity hardware experiments are quite expensive and no such facilities were available to carry out human demonstrations. The key equations that describe the learning process are reproduced from Shyam et al. (2019) as follows: the trajectories are assumed to be independent and identically distributed Gaussian with a mean and co-variance matrix.

(1) $$\begin{array}{r} y_{t}=\left[\begin{array}{c} q_{t}\\ {\overset{∙}{q}}_{t} \end{array}\right]=\left[\begin{array}{c} \phi_{t}\\ {\overset{∙}{\phi }}_{t} \end{array}\right]w+\epsilon_{y} \end{array}$$

(2) $$\begin{array}{r} p\left(\tau |w\right)=\underset{t}{\prod }N\left(y_{t}|\phi_{t}w,\Sigma_{y}\right) \end{array}$$

where *y*_*t*_ represent the robot state at time *t* [angular position (*q*_*t*_) and angular velocity (${\overset{∙}{q}}_{t}$)], ϕ_*t*_ is radial basis function, *w* is the weight vector, and ϵ_*y*_ is the error in the observations with zero mean and variance Σ_*y*_. After collecting enough demonstration data, the weight vector *w* can be found out by using a ridge regression.

The demonstrations can be carried out offline in a high fidelity simulator (for instance, MuJoCo, Todorov et al., 2012) and can be used to find out the parameters of the Gaussian. Since simulators assume ideal conditions, it is quite important to monitor the performance in actual operating conditions. Hence, the variability arising due to unforeseen circumstances can be mitigated using the incremental learning algorithm 1 proposed here. Usually, targets coordinates are specified in task space. Hence, inverse kinematics is needed to convert the task space co-ordinates to joint space parameters of the robot. It is known that a Gaussian can be conditioned to pass through desired observations, as shown in Equation (3).

(3) $$\begin{array}{l} \mu_{w}^{\left[cond\right]}=\mu_{w}-new+L(x_{t}^{*}-\phi_{t}{}^{T}\mu_{w}-new)\\ \Sigma_{w}^{\left[cond\right]}=\Sigma_{w}-new-L\phi_{t}{}^{T}\Sigma_{w}-new \end{array}$$

where *L* is

(4) $$L=\Sigma_{w}-new\phi_{t}{\left(\Sigma_{x}^{*}+\phi_{t}{}^{T}\Sigma_{w}-new\phi_{t}\right)}^{-1}$$

and $\Sigma_{x}^{*}$ is the desired accuracy to which the desired state ($x_{t}^{*}$) is to be reached. So the whole trajectory learning and adaptation can be summarized as follows:

- Incrementally learn the parameters of the Gaussian as illustrated in Algorithm 1,
- Get the target coordinates from the vision system and convert it into joint space parameters of the robot,
- Sample out trajectories from Gaussian and condition it (Equation 3) to reach the target.

Shyam et al. (2019) also provide a comparison of motion planning using learning from demonstrations against conventional optimization methods and discusses the advantages of the proposed method.


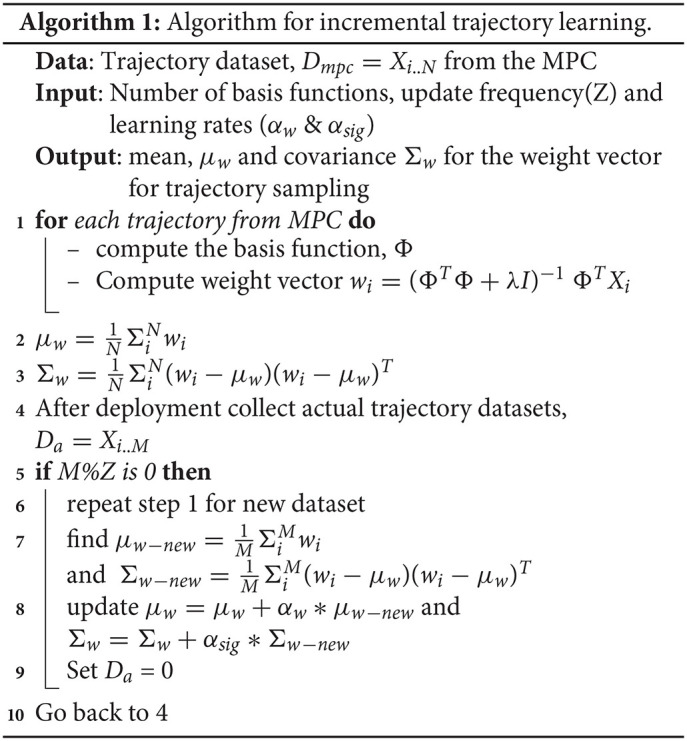


## Testbed Setups and the Demonstration Mission

### The Testbeds

**Figure 7:**
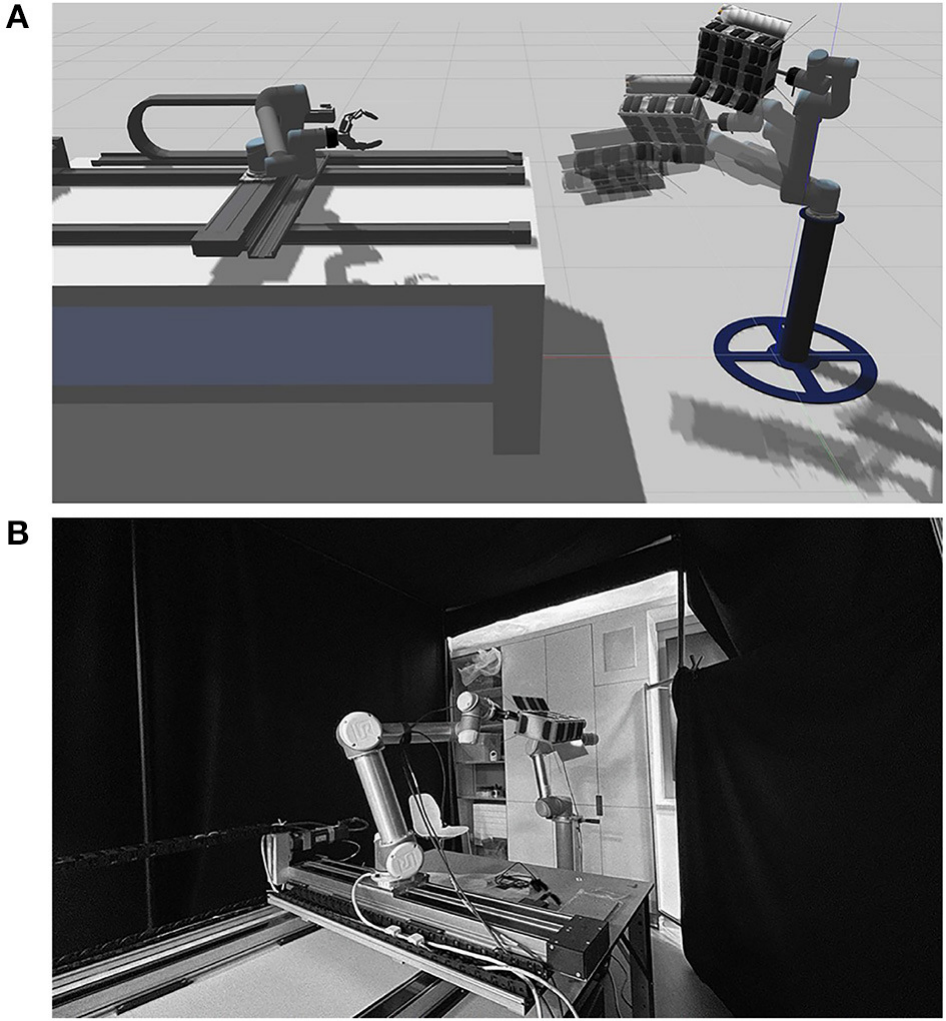
The STAR-LAB's testbeds for orbital space robotic Guidance, Navigation, and Control (GNC) systems **(A)** is the digital simulation testbed build in ROS gazebo environment; **(B)** the design and setup configuration of the STAR-LAB orbital robotic testbed in monochrome format is shown.

We have developed two testbeds for testing the proposed AI GNC system- digital and physical to demonstrate the intelligent visual GNC system. The digital tested, as shown in Figure 7A, is developed in the Robot Operating System (ROS) framework with Gazebo dynamic simulator. The collection of ROS packages can simulate sensors and two robotic arms simultaneously for fast and safe benchmarking of the proposed GNC system. The Gazebo setup acts as a digital twin to the physical testbed, as shown in Figure 7B. The physical testbeds located at the STAR LAB are the third-generation space robotic testbed that uses multi-DOF robotic arms and a 2-DOF traverser to simulate the relative motions between the service spacecraft and the target (Hao et al., 2019).

The physical testbed consists of two UR5 robotic arms—the service arm and the target arm. The service arm sits on top of a 2-DOF traverser, demonstrating the longer approaching or berthing motion between the service spacecraft and the target. The target arm offers the 6-DOF motion capability to off-load and drive the target mounted as the end-effector to follow the desired trajectory and pose as in space. The space lighting condition in the digital testbed is realized by channeling the same relative pose information to the graphic rendering engine in a synchronized link. The high-fidelity space lighting condition is demonstrated in the physical hardware testbed using a light-absorbing 45% polyacrylic + 55% cotton fabric as shown as the background in Figure 7B and halogen lamp with the sunlight spectrum and the proper brightness. The illuminance (lux) of the light source is less relevant as it is a thermal critical property. Taking advantage of the pre-calibration of the hardware locations and pose of the physical testbed as well as using the ROS transfer function broadcaster with factorial calibrated Unified Robot Description Format (URDF), the whole setup can provide ground truth reference of the position and orientation of the target and the end-effector of the service arm. Besides, the setup is implementing the Qualysis 301 camera systems for high-precision tracking.

One of the major benefits of using the ROS framework is the industrial driver support and control solutions for interactive robotic arms like UR5. The control interface offers trajectory or velocity tracking for the target arm. Using this, the CubeSat target is controlled to follow the desired trajectory or velocity as free-flying in orbits. For circular orbits in this demo mission, the desired relative trajectory or velocity waypoints can be estimated by the Clohessy and Wiltshire (CW) equations. For elliptical orbits, the Tschauner and Hempel (TH) equations provide the solution. Hao et al. (2019) provides more information about the STAR LAB's third-generation orbital robotics tested. The testbeds will be used to evaluate the GNC system with real-time and hardware-in-the-loop capability.

### The Demonstration Simulation

The demonstration mission is conceived for on-orbit manipulating and servicing a malfunctioning 16U CubeSat in low Earth orbits. This research only focuses on the intelligent visual GNC demonstration in the final approaching and close-approximate manipulation range without the initial approach and post-capture servicing phases. The starting orbits of both spacecraft for the final approach and close-approximate manipulation are circular sun-synchronous orbit with 10 s Local Time of Ascending Node (LTAN) apart as listed in Table 2.

**Table 2:** Orbital properties of the service spacecraft and the target 16U CubeSat for the demonstration mission.

	**Circular orbit altitude (km)**	**Inclination (^°^)**	**LATAN (h)**
Service spacecraft	599.95	98	10:30:00
Target 6U CubeSat	600	98	10:29:50

#### The Intelligent Target Identification and Pose Estimation

**Figure 8:**
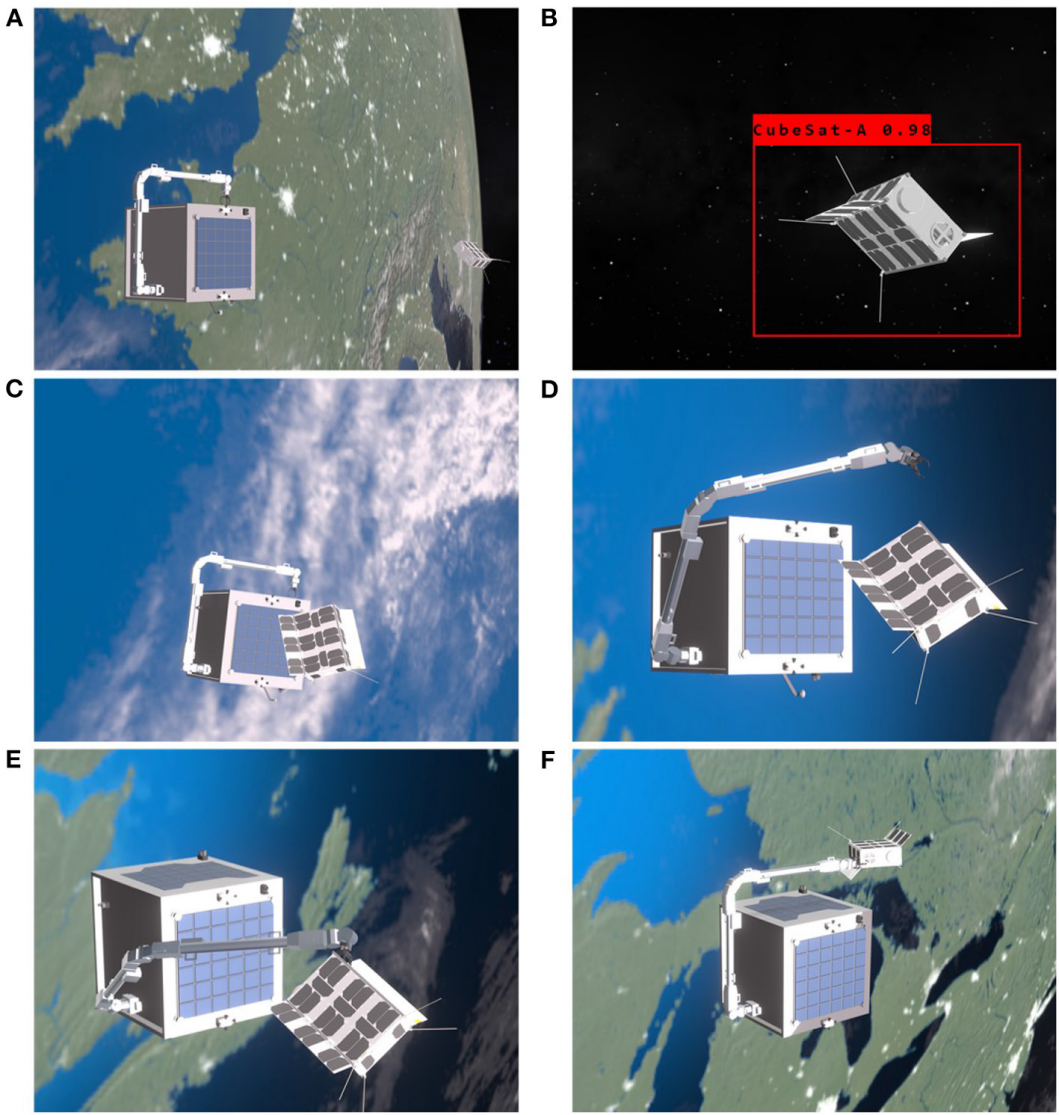
The simulation of the intelligent orbital Guidance, Navigation, and Control (GNC) operations: **(A)** the starting position of the simulation, **(B)** target identification and continuously pose estimation of the target within the camera array range, **(C)** final approaching with adjusted orbital and attitude control to an idea manipulation-ready state, **(D)** initiate the planned optimized trajectory to dock the end-effector to the pre-grasping point, **(E)** the end-effector arrives the dedicated located and executing grasping and rigidization, **(F)** plan and move the target CubeSat to for servicing. The animation is rendered in real-time in the Eevee engine with the actual relative position of the service spacecraft and the target, robotic arm trajectory points with kinematics, and time-synchronized camera readings in the render engine.

**Figure 9:**
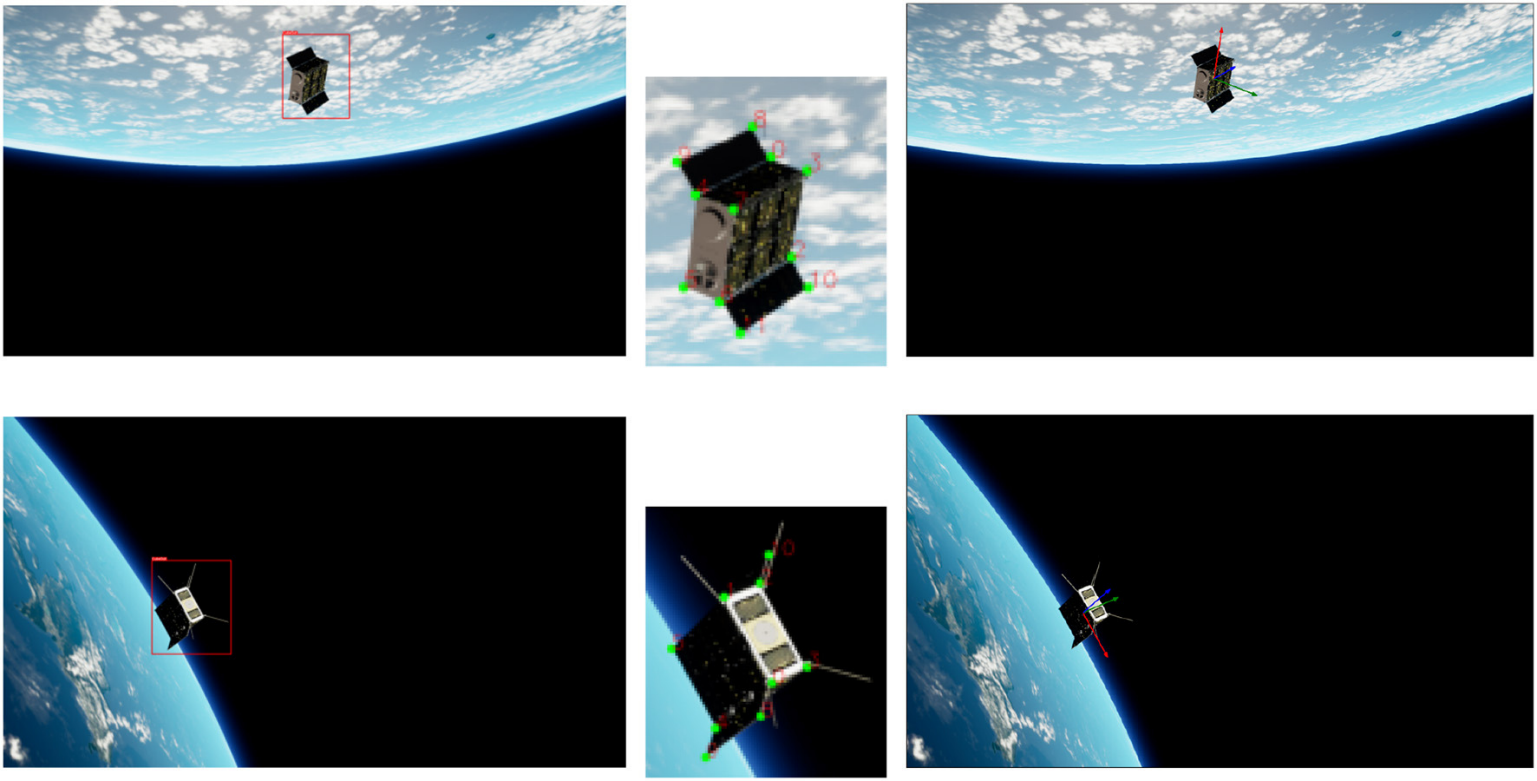
Results from the trained model (both object detection and keypoint estimation) along with computed pose from PnP.

The Intelligent Space Camera Array System (ISCAS) plays an essential role throughout the GNC algorithms of on-orbit manipulation. Figure 8A is the starting position of the two orbiting vehicles that are far apart initially. The mission starts when the target CubeSat appears in the ISCAS as a potential object. The pre-trained neural network on-board is actively determining the object and if the object is known, then it carries out a search in the onboard database for some basic information of the object. As introduced in section The Intelligent GNC System Design„ the valuable information for the intelligent GNC system includes orbital information for relative navigation, health and recent housekeeping data, and any potential service requirement for the target. Figure 8B illustrates that the ISCAS keeps working on the target identification and pose estimation. This CV process should be active during the flight mission, thus requiring efficient CV algorithms and tensor cores. Figure 9 shows the detailed simulation result of the target identification and pose estimation by using the proposed method. The target CubeSat is identified and highlighted in the red frame and the keypoints are labeled. The keypoints are then used to locate the origin of the target and generate pose information.

**Figure 10:**
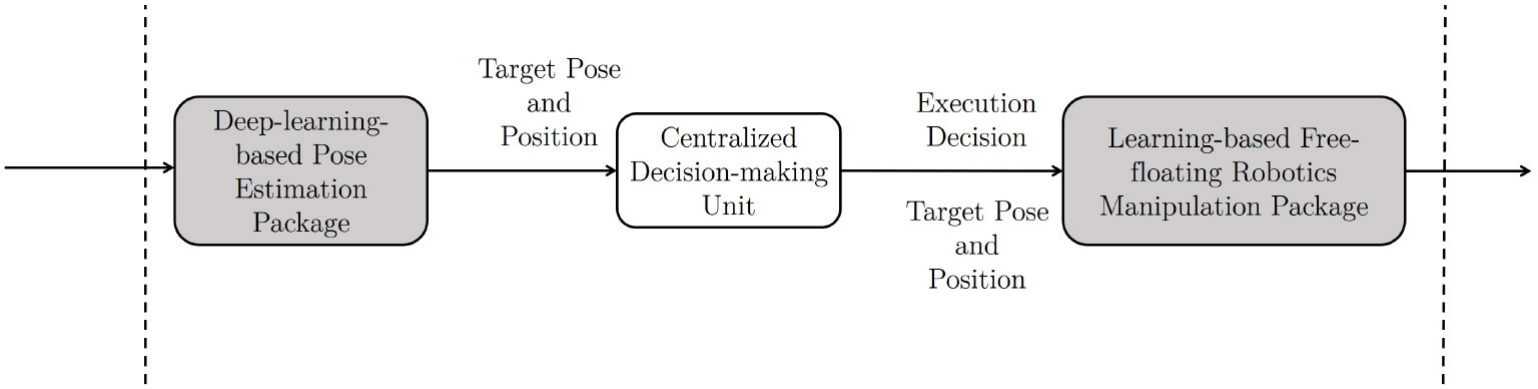
The two aforementioned Artificial Intelligence (AI) packages are connected through a decision-making block, which can use a conventional method as an intermediate solution.

As for the intelligent GNC solution proposed in this research, the packages act as the front-bone for the spacecraft rendezvous and docking to provide mid- to long-range target identification, close-proximity range pose, and position information of the target. For mid- and long-range orbit rendezvous in this intelligent approach, it is recommended to use the conventional approaches as an intermediate solution. Hence, for low Earth orbits like the mission demonstrated here, the conventional in-front rendezvous with a higher, more elliptical orbit for the service spacecraft is preferred for simplicity. However, as the novel AI-based approach introduced here is a transitional approach toward full autonomy, this mission is designed to use the back and lower circular orbit rendezvous, which usually provides a better safe margin at the cost of slower approaching. Furthermore, from the concept demonstration, both the target and the service spacecraft have circular orbits that enable the testbeds to implement the C-W equations to deliver high-fidelity relative motion. A decision-making link is required between the pose estimation and the manipulator control modules. As shown in Figure 10, the demonstration uses a centralized decision-making unit that could be either handled by the conventional remote operations or onboard logic unit.

#### The Learning-Based Free-Floating Manipulator Control

**Figure 11:**
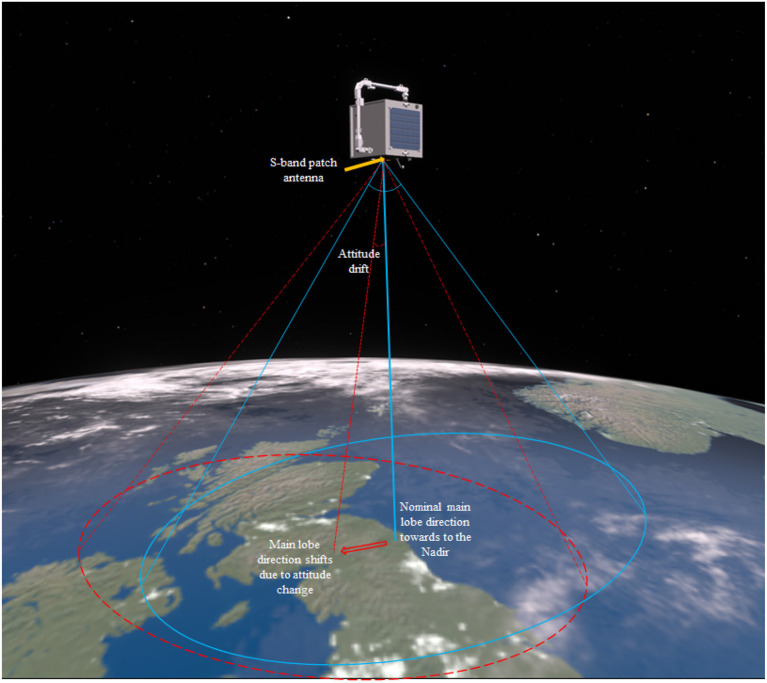
The attitude requirement for proposed free-floating spacecraft is to minimize the angular drift within the half-power beamwidth (HPBW) during manipulation period to maintain stable communication.

Once the rendezvous and velocity matching are finished, the multi-DOF robotic arm is executed to move the end-effector to the target position in the close-proximity. Figures 8C,D shows the actuation of the robotic arm following the optimized trajectory while maintaining the minimal rotation to the service spacecraft in the free-floating mode. The attitude requirement for this demonstration is to minimize the attitude drift away from the nominal flying condition, at which the S-band patch antenna pointing toward to the Nadir. The maximum attitude drift should remain within the half-power beamwidth (HPBW) lobe, which is a bubble shape and has a HPBW of ±50° as shown in Figure 11 for this particular S-band patch antenna. This attitude range can ensure the spacecraft remains stable communication link with the ground station even during the manipulation. As mentioned before, conventional probabilistic robotic arm planning methods cannot control the free-floating platform as the dynamics of the free-floating base is coupled with the movement of the robotic arm. To date, very limited number of research has investigated the coupled dynamics and kinematics problem (Nanos and Papadopoulos, 2017) and the planning methods are expensive and difficult to be used for the free-floating spacecraft attitude optimization with high-DOF robotic arms. In addition, the monocular cameras cluster system, which has 360° coverage, has no attitude pointing margin as needed for the conventional Attitude Determination and Control System (ADCS) sensors. For example, spacecraft with star tracker needs to avoid direct sunlight toward the sensor.

**Figure 12:**
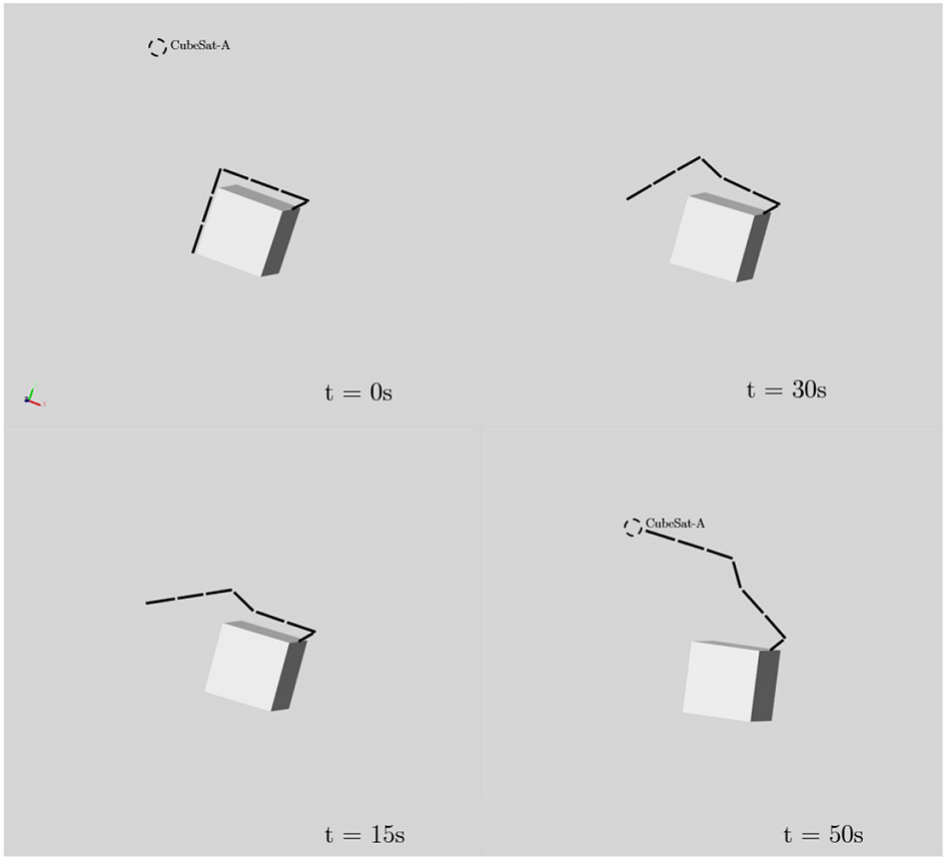
Time series of the free-floating spacecraft reaching the CubeSat position with the trajectory sampled from learned probabilistic distribution. This trajectory has minimized impact to the attitude of the free-floating spacecraft.

**Figure 13:**
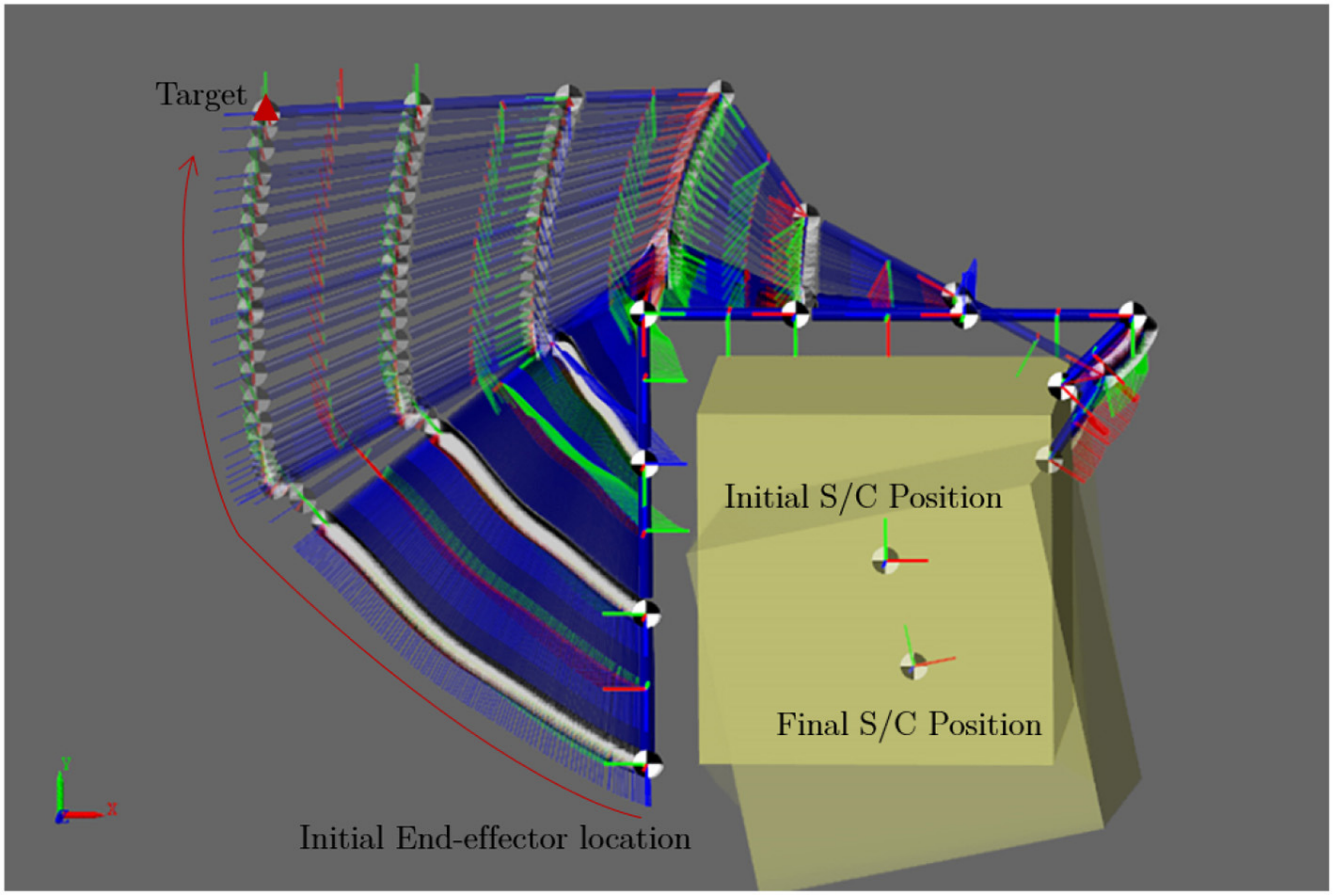
Motion trajectory sampled from learned probabilistic distribution to minimize the rotation of the free-floating spacecraft.

The following dynamic simulation result shows the motion of the 7-DOF manipulator and the free-floating spacecraft giving the trajectory generated by the proposed learning-based planning method. The dynamic simulation carried out in MATLAB-SIMULINK environment and the result is shown in Figure 12 with the relative velocity between the target and the spacecraft is zero at the beginning of the simulation. The URDF file and optimized trajectory generated by the learning-based method are provided as available data along with this paper. Note that the URDF differs from the AISAT concept design shown in the rendering because the URDF file is customized for the offline trajectory sampling. The same file is also used for the dynamic simulation carried out in MATLAB-SIMULINK to evaluate the free-floating spacecraft attitude drift. However, the Eevee engine uses the modified trajectory and model from the URDF file to demonstrate the concept in the rendering. Figure 13 presents the optimized trajectory from the proposed method with unified time interval. It shows a relative rapid motion in the beginning, and then followed by a slower arm movement while the overall moment of inertia of the system is increasing due to the extension of the 7-DOF arm. The supplementary animation shows how this trajectory tries to maintain the spacecraft attitude when it controls the 7-DOF manipulator. The bottom level controller used for this dynamic simulation is a centralized PID controller for all joints. Supplementary Material with this article also includes the spline refined joint space array of the trajectory to reproduce the result.

**Figure 14:**
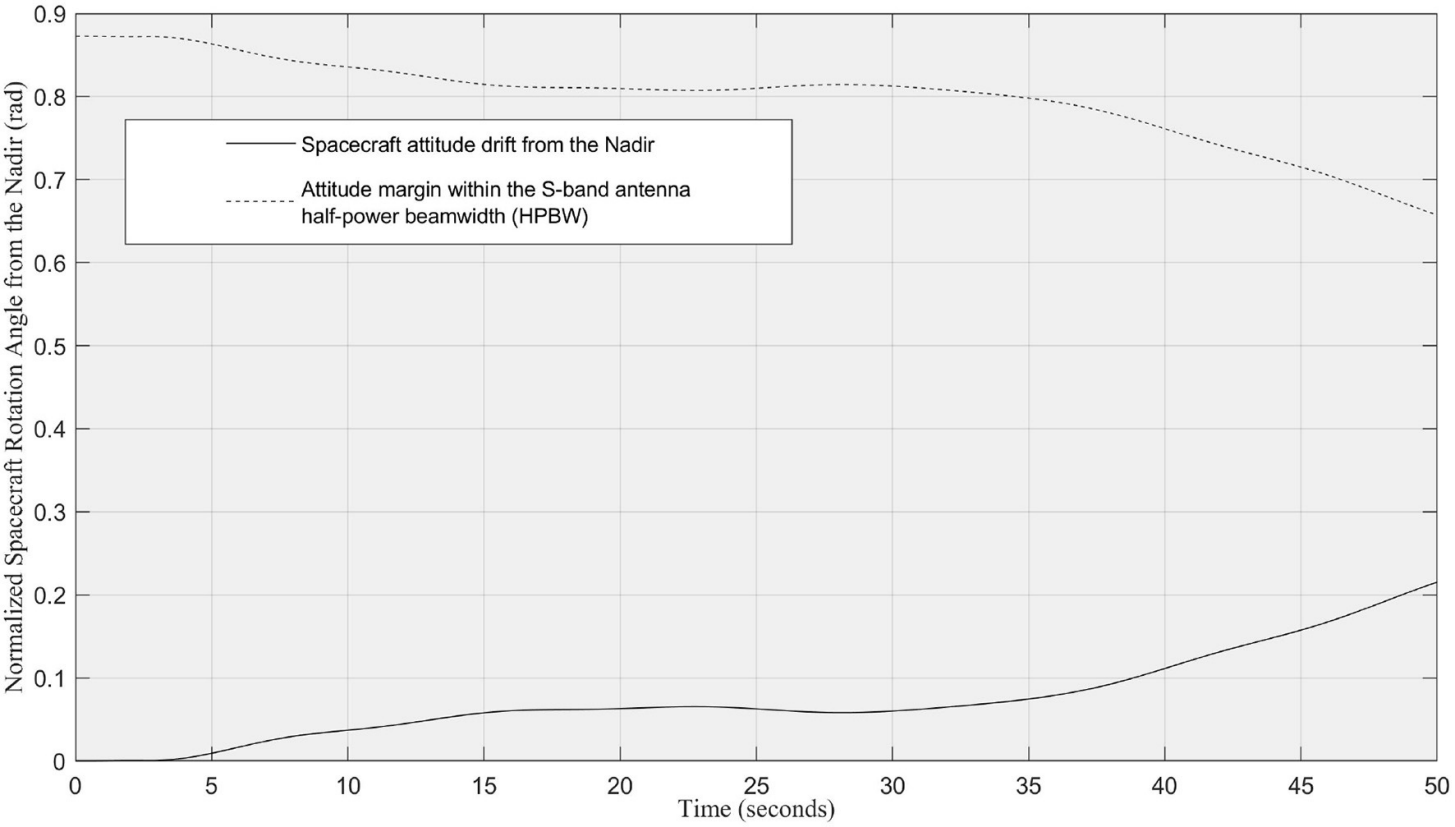
The normalized attitude drift (in rads) of the free-floating spacecraft relative to the Nadir during the manipulator operation in the simulation. The attitude drift is within the S-band patch antenna half-power beamwidth (HPBW) for stable ground communication.

Figure 14 shows the spacecraft attitude change during the manipulation. By considering the attitude pointing requirement for the S-band patch antenna HPBW, the result shows an overall high margin in the attitude requirement to maintain a high-quality communication link. This result implies that the learning-based trajectory planning method is a viable solution to solve the complex high-DOF dynamic and kinematic planning problem with attitude optimization. Finally, Figures 8E,F illustrates the grasping phase and the pose-capture servicing phase, respectively. Those two phases are out of the scope of this paper. Ideally, grasping and pose-grasping require the service spacecraft to actively stabilized its attitude and to damp the impact oscillation in order to reduce the collision risk.

### Benefits and Limitations

The benefits of using the proposed AI enhanced GNC system for future space missions are listed as follow:

- Increased GNC autonomy: The proposed system could potentially allow future spacecraft to use 2D images to identify and determine the pose of the orbiting targets in real time for autonomous navigation and control. However, the conventional GNC system usually requires the cooperation from the target or ground analysis before executing the navigation and control.
- Low-cost visual sensors: The proposed method only uses monocular cameras for the GNC system. Space qualified monocular cameras are relative inexpensive and have a solid flight heritage. They are also easy to use and less power hungry than other potential 3D sensors like LiDAR.
- Manipulation trajectory planning with free-floating spacecraft attitude constraint: The proposed method of sampling the trajectory of the manipulator with complying attitude requirements could address the problems of the spacecraft attitude drifts, which is considered as a major problem of using free-floating spacecraft for space robotic operations. Conventional trajectory planning methods require computationally expensive optimization problem to be solved for the multi-DOF robotic arm on a free-floating platform. This will be difficult for onboard computers.

This novel GNC system with the emerging AI components could potentially increase the reliance on software as opposed to hardware. This method could be implemented on increasingly complex space missions in future without the need to introduce expensive and novel hardware. The AI software can be potentially implemented with software updates and reduce the mission cost as hardware R&D and tooling, which are the expensive barriers for the space industry.

Nevertheless, using AI for spacecraft with manipulator comes with many costs and introduces new challenges to the traditional spacecraft mission design and flight software Verification and Validate (V&V), such as risk control, computational power, and system stability. Although AI algorithms have been widely adopted for CV and data science applications where the end-user is human, those algorithms are still considered as high risk for industrial engineering applications, particularly for the space industry that has extremely low-risk tolerance. However, we believe the trustworthiness and reliability will be gradually improved with more research and technology demonstrations being carried out in the future. One of the methods can be used to address the design stability issue for the AI system is to define a safe threshold for each of the GNC components. For example, during the pose estimation, we can set a higher threshold for object detection and keypoint estimation, through which lower accuracy estimations can be filtered out to maintain more reliable estimated poses. Another risk associated with the orbital manipulation is collision. Once we estimate the target's relative pose, since it is a known target one can easily perform collision avoidance by using the existing target CAD models. In addition, the proposed method needs the onboard computer to effectively implement and execute the AI algorithms, which usually demand high computing power. This challenge is soon likely to be addressed from the recent research initiated by ESA and NASA to develop radiation-proof high power computing unit and immigrate existing AI packages into the existing space qualified computer (Lentaris et al., 2018; ESA, 2019a).

## Conclusion and Future Works

This research shows a preliminary exploration and an overview of the feasibility of using emerging machine-learning techniques for space missions that involve on-orbital manipulation, which is essential for on-orbit service missions. The novel intelligent GNC system proposed in this paper aims to be an intermediate solution between the conventional spacecraft GNC architecture and the fully intelligent system that can utilize all sensor information to independently plan and execute GNC tasks to achieve meaningful objectives for on-orbit servicing. The proposed system can potentially boost the autonomy of the spacecraft GNC system for autonomous on-orbit target manipulation. The core concept of the intelligent GNC system is using the centralized camera network solely to feed 2D images of the target into the pre-trained neural network for target identification, pose estimation, and then use another learning-based method to plan the multi-DOF manipulator to capture the target while minimizing the drift of the free-floating spacecraft from its nominal flying attitude. As the system uses target identification with known targets database searching for long- and mid-range relative navigation, the proposed intelligent GNC system serves as a bridge to offer limited onboard intelligence for task planning and decision-making. The use case is developed around an on-orbit servicing mission for a 16U CubeSat as the known target to demonstrate the concept. The outline of the mission with the simulation results of each of the core components of the proposed intelligent GNC system are presented and discussed to show the system's feasibility and potential at the conceptual level.

The common limitations and concerns of using AI algorithms for engineering applications inevitably apply to space missions as well, such as V&V, reliability, and explainability. To overcome the trustworthiness issues, more hardware-in-the-loop testings and demonstrations with actual engineering implementations are necessary. The testbeds developed for this research are also presented in this paper. They will be used to verify and showcase the GNC system to increase the TRLs, and hopefully discover potential avenues for research. Another possible future agenda is to consider reinforcement learning methods that have shown some promising results for “visual to actuation” as a potential step toward the fully intelligent GNC system for on-orbit servicing.

## Data Availability Statement

The datasets presented in this study can be found in online repositories. The names of the repository/repositories and accession number(s) can be found in the article/Supplementary Material.

## Author Contributions

ZH conceived the idea of the intermediate solution of using AI components for future spacecraft visual GNC system and designed the spacecraft system and mission, implemented the simulation and generated the physics-based rendering for the demonstration mission, integrated and implemented the bottom-level controller, gathered the simulation results and data analysis, and also carried out the team coordination, major literature survey, and main manuscript writing. RS conceived the idea of offline robot trajectory generation by learning from demonstrations and reproduction by sampling and conditioning of the learned probabilistic distribution, and provided the optimized trajectory for the simulation by developing a dynamic model in URDF format, and also conceived the incremental learning Algorithm 1, lead the section Learning-Based Method For Robotic ARM Manipulation and Control writing, and helped with literature survey and overall manuscript writing. AR conceived the idea of the novel DL pose estimation algorithm and contributed to the DL data generation and training, and the pose estimation demonstration, lead the section Deep-Learning Method For Target Pose Estimation writing, and contributed to literature survey. YG was the research group lead and offered the supervision to this research topic. All authors contributed to the article and approved the submitted version.

## Conflict of Interest

The authors declare that the research was conducted in the absence of any commercial or financial relationships that could be construed as a potential conflict of interest.

## References

[B1] Boumans R. Heemskerk C. (1998). The European robotic arm for the international space station. Robot. Auton. Syst. 23, 17–27. 10.1016/S0921-8890(97)00054-715008205

[B2] Chen B. Cao J. Parra A. Chin T. J. (2019). “Satellite pose estimation with deep landmark regression and nonlinear pose refinement,” in 2019 IEEE/CVF International Conference on Computer Vision Workshop (ICCVW) (Seoul: IEEE), 2816–2824. 10.1109/ICCVW.2019.00343

[B3] Cheng B. Xiao B. Wang J. Shi H. Huang T. S. Zhang L. (2020). “HigherHRNet: scale-aware representation learning for bottom-up human pose estimation,” in CVPR (Seattle, WA), 1–10. 10.1109/CVPR42600.2020.00543

[B4] Chien S. Jonsson A. Knight R. (2005). Automated Planning & Scheduling for Space Mission Operations. Jet Propulsion Laboratory Available online at: https://trs.jpl.nasa.gov/bitstream/handle/2014/41515/05-0533.pdf?sequence=1 (accessed on September 01, 2020).

[B5] Dubowsky S. Papadopoulos E. (1993). The kinematics, dynamics, and control of free-flying and free-floating space robotic systems. IEEE Trans. Robot. Autom. 9, 531–543. 10.1109/70.258046

[B6] ESA (2019a). AIKO: Autonomous satellite operations thanks to Artificial Intelligence. ESA.

[B7] ESA (2019b). Kelvins–ESA's Advanced Concepts Competition. ESA. Available online at: https://kelvins.esa.int/satellite-pose-estimation-challenge/ (accessed on January 06, 2020).

[B8] Forshaw J. L. Aglietti G. S. Navarathinam N. Kadhem H. Salmon T. Pisseloup A.. (2016). Removedebris: an in-orbit active debris removal demonstration mission. Acta Astron. 127, 448–463. 10.1016/j.actaastro.2016.06.018

[B9] Gerard K. (2019). Segmentation-Driven Satellite Pose Estimation. Technical Report. EPFL. Available online at: https://indico.esa.int/event/319/attachments/3561/4754/pose_gerard_segmentation.pdf

[B10] Goodfellow I. Bengio Y. Courville A. Bengio Y. (2016). Deep Learning, Vol. 1. Cambridge, MA: MIT Press.

[B11] Hao Z. Mavrakis N. Proenca P. Darnley R. G. Fallah S. Sweeting M.. (2019). “Ground-based high-DoF AI and robotics demonstrator for in-orbit space optical telescope assembly,” in Proceedings of the International Astronautical Congress (Washington, DC: IAC).

[B12] Lentaris G. Maragos K. Stratakos I. Papadopoulos L. Papanikolaou O. Soudris D.. (2018). High-performance embedded computing in space: evaluation of platforms for vision-based navigation. J. Aerosp. Inform. Syst. 15, 178–192. 10.2514/1.I010555

[B13] Nanjangud A. Blacker P. C. Bandyopadhyay S. Gao Y. (2018). Robotics and AI-enabled on-orbit operations with future generation of small satellites. Proc. IEEE 106, 429–439. 10.1109/JPROC.2018.2794829

[B14] Nanos K. Papadopoulos E. G. (2017). On the dynamics and control of free-floating space manipulator systems in the presence of angular momentum. Front. Robot. AI 4:26. 10.3389/frobt.2017.00026

[B15] Opromolla R. Fasano G. Rufino G. Grassi M. (2017). A review of cooperative and uncooperative spacecraft pose determination techniques for close-proximity operations. Prog. Aerosp. Sci. 93, 53–72. 10.1016/j.paerosci.2017.07.001

[B16] Pasqualetto Cassinis L. Fonod R. Gill E. (2019). Review of the robustness and applicability of monocular pose estimation systems for relative navigation with an uncooperative spacecraft. Prog. Aerosp. Sci. 110:100548. 10.1016/j.paerosci.2019.05.008

[B17] Pierson H. A. Gashler M. S. (2017). Deep learning in robotics: a review of recent research. Adv. Robot. 31, 821–835. 10.1080/01691864.2017.1365009

[B18] Proenca P. F. Gao Y. (2020). “Deep learning for spacecraft pose estimation from photorealistic rendering,” in 2020 IEEE International Conference on Robotics and Automation (ICRA) (Paris: IEEE), 6007–6013. 10.1109/ICRA40945.2020.9197244

[B19] Rathinam A. Gao Y. (2020). “On-orbit relative navigation near a known target using monocular vision and convolutional neural networks for pose estimation,” in International Symposium on Artificial Intelligence, Robotics and Automation in Space (iSAIRAS), Virutal Conference (Pasadena, CA), 1–6.

[B20] Sharma S. D'Amico S. (2019). “Pose estimation for non-cooperative rendezvous using neural networks,” in AIAA/AAS Space Flight Mechanics Meeting (Big Sky, MT). 10.1109/AERO.2018.8396425

[B21] Shyam R. Lightbody P. Das G. Liu P. Gomez-Gonzalez S. Neumann G. (2019). “Improving local trajectory optimisation using probabilistic movement primitives,” in International Conference on Intelligent Robots and Systems (IROS) (Macau: IEEE). 10.1109/IROS40897.2019.8967980

[B22] Starek J. A. Açıkmeşe B. Nesnas I. A. Pavone M. (2016). Spacecraft Autonomy Challenges for Next-Generation Space Missions. Berlin; Heidelberg: Springer Berlin Heidelberg.

[B23] Tipaldi M. Glielmo L. (2018). A survey on model-based mission planning and execution for autonomous spacecraft. IEEE Syst. J. 12, 3893–3905. 10.1109/JSYST.2017.2720682

[B24] Todorov E. Erez T. Tassa Y. (2012). “Mujoco: a physics engine for model-based control,” in 2012 IEEE/RSJ International Conference on Intelligent Robots and Systems (Vilamoura-Algarve: IEEE), 5026–5033. 10.1109/IROS.2012.6386109

[B25] Truszkowski W. Hallock H. Rouff C. Karlin J. Rash J. Hinchey M.. (2009). Autonomous and Autonomic Systems: With Applications to NASA Intelligent Spacecraft Operations and Exploration Systems. Springer Science & Business Media. 10.1007/b105417

[B26] Umetani Y. Yoshida K. (1989). Resolved motion rate control of space manipulators with generalized Jacobian matrix. IEEE Trans. Robot. Autom. 5, 303–314. 10.1109/70.34766

[B27] Virgili-Llop J. Romano M. (2019). Simultaneous capture and detumble of a resident space object by a free-flying spacecraft-manipulator system. Front. Robot. AI 6:14. 10.3389/frobt.2019.00014 33501030 PMC7805840

[B28] Wilde M. Kwok Choon S. Grompone A. Romano M. (2018). Equations of motion of free-floating spacecraft-manipulator systems: an engineer's tutorial. Front. Robot. AI 5:41. 10.3389/frobt.2018.00041 33500927 PMC7806027

